# Divergence in life-history traits among three adjoining populations of the sea snake *Emydocephalus annulatus* (Hydrophiinae, Elapidae)

**DOI:** 10.1038/s41598-022-09130-y

**Published:** 2022-03-24

**Authors:** Richard Shine, Gregory P. Brown, Claire Goiran

**Affiliations:** 1 School of Natural Sciences, Macquarie University, Sydney, NSW 2109 Australia; 2 School of Life and Environmental Sciences, University of Sydney, Sydney, NSW 2006 Australia; 3 LabEx Corail & ISEA, Université de La Nouvelle-Calédonie, BP R4, 98851 Nouméa cedex, New Caledonia

**Keywords:** Behavioural ecology, Conservation biology, Evolutionary ecology, Population dynamics, Tropical ecology

## Abstract

Life-history traits such as rates of growth, survival and reproduction can vary though time within a single population, or through space among populations, due to abiotically-driven changes in resource availability. In terrestrial reptiles, parameters such as temperature and rainfall generate variation in life-histories—but other parameters likely are more important in marine systems. We studied three populations of sea snakes (*Emydocephalus annulatus*) in adjacent bays in the IndoPacific archipelago of New Caledonia. The extreme philopatry of individual snakes allows us to unambiguously allocate each animal to one of the three populations. Although water temperatures and rainfall do not differ over this small scale, one site experiences more intense winds, restricting opportunities for foraging. Our 18-year mark-recapture dataset (> 1,200 snakes, > 2,400 captures) reveals significant divergence among populations in life-history traits. Survival rates and population densities were similar among sites, but snakes at the most wind-exposed site (Anse Vata) exhibited lower body condition, slower growth, less frequent production of litters, and smaller litters. Weather-driven variation in feeding rates thus may affect life-history traits of marine snakes as well as their terrestrial counterparts, but driven by different parameters (e.g., wind exposure rather than variation in temperatures or rainfall).

## Introduction

Rates of survival, growth and reproduction are critical determinants of variation in individual lifetime reproductive success, and hence are expected to be under strong natural selection^1^. Although mathematical models predict that intense selection for an optimal value of some trait (such as reproductive frequency or litter size) should erode variation in that trait, life-history characteristics often vary considerably through both space and time within a single species^2,3^. The resolution of this paradox lies in the fact that rates of energy expenditure on specific life-history pathways are constrained by the need to acquire resources (energy) for reproduction, preventing individual organisms from growing and reproducing at optimal levels. The dependence of life-history traits on local resource availability is most apparent in sessile organisms such as plants, in which the growth trajectory and reproductive output of an individual is constrained by access to water, light and nutrients^4^. By dispersing among sites, mobile organisms reduce the impact of spatial variability in resource levels (by averaging over a larger area) but nonetheless often exhibit substantial variation in life-histories over broader geographic comparisons (e.g., tropical vs. temperate; desert vs. oasis; mainland vs. island^5,6^) as well as through time at a single site^7,8^.

As the example of plants demonstrates, the most powerful systems in which to explore the impact of habitat heterogeneity on life-history traits involve organisms that are sessile, or at best highly philopatric. In such a species, an individual experiences conditions at the same site throughout its life; and thus, spatial variation in factors such as resource availability should generate spatial variation in life-history attributes. In reptiles, most research on the drivers of variation in life-history involve analyses through time at a single site (weather effects^8,9^) and broad-scale comparisons of populations inhabiting widely-separated areas with different climatic conditions (e.g., tropical-temperate^10–12^). Nonetheless, some studies have documented cases where adjacent habitats differ in ways that cause life-history divergences either as direct effects or via adaptation^13,14^.

Studies of intraspecific variation in life-history attributes of reptiles have focused on terrestrial and freshwater species, reflecting the logistical difficulties of studying marine animals (but for analyses of sea turtles see^15–18^). In terrestrial snakes, many researchers have documented spatiotemporal shifts in life-history characteristics, often related to variation in resource availability^8,9,19^. In contrast, sea snakes have attracted less attention, with longterm mark-recapture studies rarely conducted^20^. Our 18-year study on a highly philopatric sea snake species provides an opportunity to examine whether or not life-history traits differ among populations from adjacent sites.

## Materials and methods

### Study species

**Figure 1:**
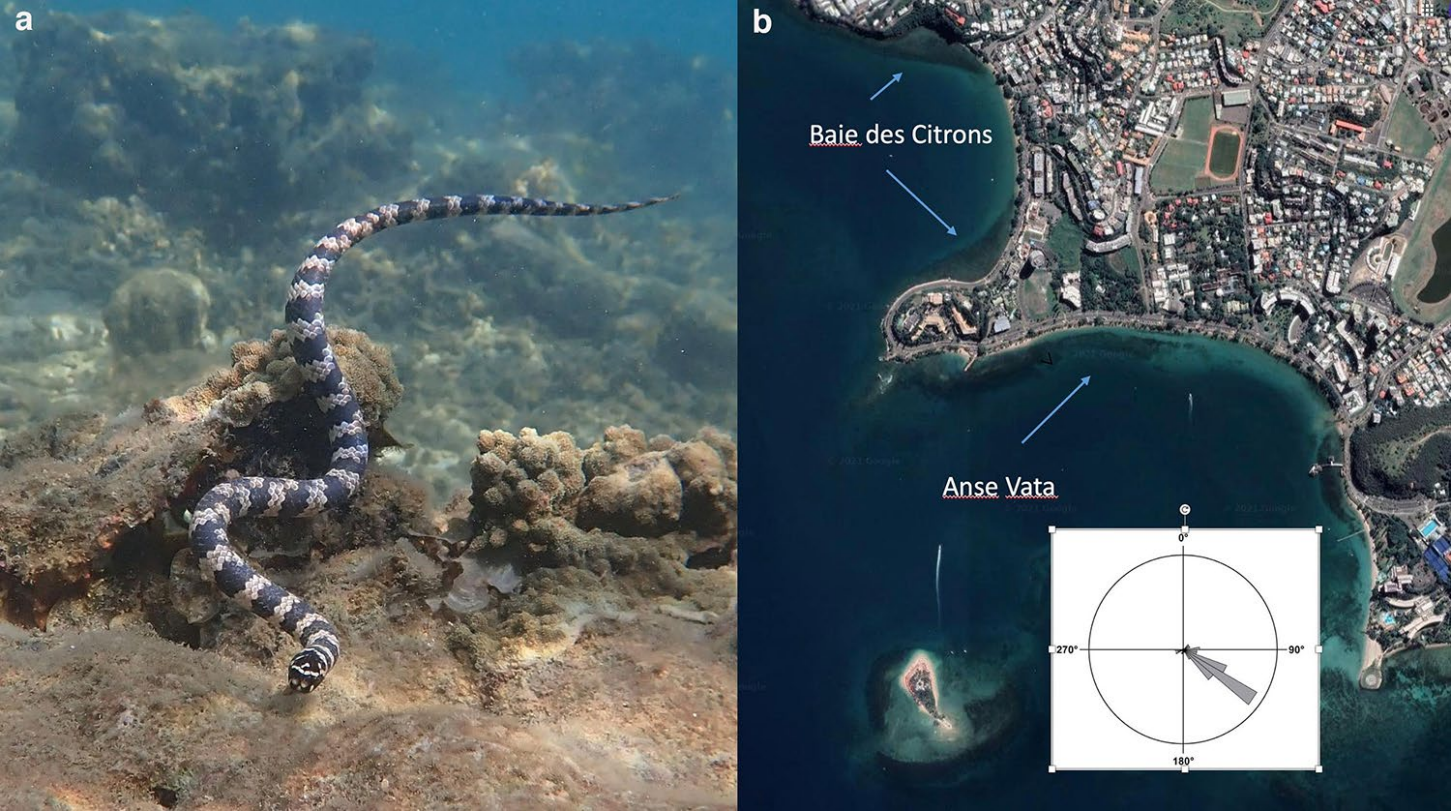
Turtle-headed sea snake *Emydocephalus annulatus* (**a**) and the location of the three study sites used (**b**). On the aerial photograph, areas of coral reef habitat are darker than adjacent sandy sites; the arrows point to reef areas at Anse Vata, and on the northern and southern ends of the Baie des Citrons. The inset diagram shows the compass direction from which winds blow, based on monthly mean data, showing that Anse Vata is more exposed than the Baie des Citrons. Photograph (**a**) by Claire Goiran. Photograph (**b**) modified from https://earth.google.com/web/@-22.29399082,166.44822979,20.67054793a,11283.26626082d,35y,0h,0t,0r.

Widely distributed through shallow waters of northern Australia through to the Coral Sea, turtle-headed sea snakes (*Emydocephalus annulatus*) are fully aquatic (hydrophiine) elapid snakes^20,21^. Relatively stout-bodied, females in our study sites attain around 800 mm snout-vent length (SVL) whereas males are smaller (to 700 mm^20^; Fig. 1a). Unusually among marine snakes, this species feeds entirely on the eggs of demersal-spawning fishes^22^. As a result, these snakes have evolved to be functionally non-venomous, with small fangs and venom glands^23^.

### Study sites

The western edge of Noumea, capital city of New Caledonia, is fringed by shallow bays. We worked in two of those bays, Anse Vata and Baie des Citrons, that are separated by a rocky headland (Rocher à la voiole: 22°16′S, 166°26′E; Fig. 1b). The central part of each bay contains sandy substrates whereas the northern and southern ends of each bay harbour fringing reefs that support abundant sea snakes^24^. Our three study sites (northern end of Anse Vata, northern and southern ends of Baie des Citrons) are each around 350 × 50 m in size, extending from the water’s edge to a depth of 2–4 m. The substrate comprises a mosaic of live and dead coral, boulders, and sand (see^25^ for detailed description and analysis). Water temperatures average around 27 °C in midsummer (February) and 23 °C in midwinter (August: https://www.seatemperature.org/australia-pacific/new-caledonia/noumea.htm). Because of its orientation and lack of protection from surrounding land masses, Anse Vata is exposed to stronger winds (“trade winds”) than are the other two sites (Fig. 1b). There are no other obvious differences (in water depths, substrates, etc.) among the three sites.

Each of the three sites contains an average of around 120 turtle-headed sea snakes^21^, with approximately equal numbers of males and females (site averages 47 to 51% male). Although each site is separated from the others by less-suitable habitat (rocky or sandy rather than coral-dominated), snakes can easily move from one site to another, and one snake did so within a single day (R.S. and C.G., unpubl. data). However, migration among sites is rare (28 records from > 2,400 captures over 18 years). That extreme philopatry allows for population divergence in genetics^26^, morphology (e.g., mean body length, relative tail length, head shape), and locomotor ability^27^. The present study extends that analysis of differences among populations to include life-history traits.

### Survey methods

Every January from 2004 to 2021, we snorkelled (in groups of 3 to 12 people) through each site at least six times per year, for periods of 30–60 min each, to find and capture turtle-headed sea snakes (see^21^ for details). After being captured by hand and retained in floating cages, the snakes were taken to a nearby laboratory for measuring (SVL), weighing, scoring of sex and reproductive condition, and micro-chipping for individual identification. Sex was determined from relative tail length and scale rugosity^28^. Pregnancy and litter sizes were determined by palpation of the posterior body^20^. The animals were then released at their point of capture, usually within 1 h.

## Statistical analysis

### Wind speeds

Based on the orientation of surrounding headlands and reefs, we estimated that the study site at Anse Vata was exposed to winds from 135 to 260°, that at Baie des Citrons south from 270 to 310°, and that at Baie des Citrons north from 190 to 280° (Fig. 1b). We extracted data on wind speeds and directions from the Météo-France climate database for Noumea (http://www.meteo.nc/donnees-publiques/publitheque) and calculated seasonal means (summer = October to April; winter = May to September: see^21^) from mean monthly values. Because data for wind speeds were not normally distributed, we used Steel–Dwass nonparametric tests to compare wind speeds between pairs of sites within each season. In this and all other tests apart from survival estimates, we used JMP Pro 15.0 for analyses.

### Survival rates

Based on mark-recapture records, Cormack-Jolly-Seber estimates of survival rates for each site in each year were calculated using the POPAN implementation in the MARK software package^29^ in SAS using fully-time-dependent models, where parameters for survival were allowed to vary among sites and among years. Model selection was based on differences in corrected AIC values between contending models, with differences < 2.0 indicative of equivalent support.

### Body condition

We used residual scores from the general linear regression of ln mass against ln SVL to quantify body condition; a negative residual score indicates a snake that is thinner-than-expected based on its body length. That residual score was used as the dependent variable in an ANOVA with sex and site as factors. Year was included as a random factor, to account for pseudoreplication in time. In this and the other ANOVA and ANCOVA analyses below, Tukey post-hoc tests were used to identify significant pairwise differences.

### Growth curves

Rather than inferring ages from body sizes, we relied upon data from snakes whose ages we knew from mark-recapture data (i.e., animals first captured in their first year of life, when body size unambiguously identifies an individual as young-of-the-year^20^). We restricted the analysis to animals < 6 years of age, because of low and variable sample sizes for older age classes, and low growth rates in later life^20^. Because growth trajectories differ between the sexes^20^, we conducted analyses separately for males and for females. In each case we included site and age class as factors, plus the site*age interaction, as fixed effects in an ANOVA; year was included as a random factor.

### Reproductive frequency

For all females for which we had scored reproductive condition in at least 2 years, we calculated the proportion of years in which they were gravid when captured. We excluded females that were only scored once (i.e., captured in only one year), because their inclusion generated a highly non-random distribution of data (many zeros and ones). Because reproductive frequency increases with maternal body size^20^, we included mean SVL as a covariate in ANCOVAs with site as the factor (and site*SVL interaction), and proportion of years reproductive as the dependent variable.

### Litter sizes

We repeated the above analysis using mean litter size per female as the dependent variable, and including mean maternal SVL as a covariate. We included all adult females in this analysis.

### Ethics declaration

The research was conducted under animal ethics approval 2015/880 (University of Sydney) and permit 3252–17/ARR/DENV (Province Sud, New Caledonia). All procedures involving animals were carried out in accordance with relevant guidelines and regulations (including ARRIVE guidelines).

## Results

### Wind speeds

**Figure 2:**
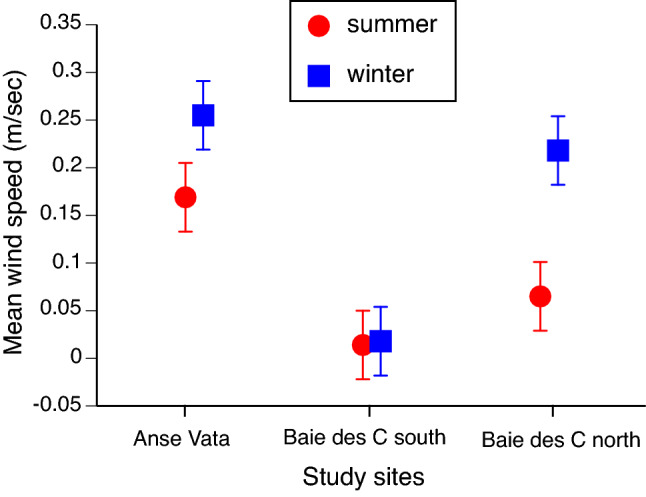
Mean wind speeds at the three study sites in summer and winter, based on orientation of the three sites with respect to prevailing wind directions combined with data on wind speeds and directions extracted from the Météo-France climate database for Noumea (http://www.meteo.nc/donnees-publiques/publitheque). Values were calculated separately for summer (October to April) and winter (May to September)^21^, based on mean monthly values. The graph shows mean values and associated standard errors.

The Baie des Citrons South site experienced calmer conditions than did the other two sites (Fig. 2). Steel–Dwass tests showed significant differences between this site and Anse Vata in both seasons (*P* = 0.003 in both cases) and between the northern and southern ends of the Baie des Citrons in winter (*P* = 0.003).

### Survival

The MARK analysis generated two top-ranking models that fit the data equally well, regardless of whether we combined data or treated the sexes separately. One of the high-ranked models allowed survival to vary over time, whereas the other had time-constant survival (in Table 1, Phi(t) and Phi(.) respectively). For both of these models, the recapture rate varied differently over time among the three sites (p(g*t) in Table 1). Using the delta AICc criterion for model selection (< 2), the top-ranked model that allowed survival rates to vary among sites (third model in Table 1, = Phi(g)) fitted the data four times less well than did the top Phi(t) model. Even under this (third-top-ranked) model, the disparity among sites in estimated annual survival rates (mean ± SE) was minor (0.67 ± 0.03 at Baie des Citrons north, 0.67 ± 0.02 at Baie des Citron south, and 0.72 ± 0.03 at Anse Vata).

**Table 1:** Ranking of Cormack-Jolly-Seber models estimating annual rates of survival (Phi) and recapture (p) of turtle-headed sea snakes (*Emydocephalus annulatus*) in three populations.

Both Sexes Pooled		
Model	AICc	Delta AICc
{Phi(t) p(g*t) PIM}	2473.2062	0
{Phi(.) p(g*t) PIM}	2474.7169	1.5107
{Phi(g) p(g*t) PIM}	2477.2338	4.0276
{Phi(g*t) p(g*t) PIM}	2479.8951	6.6889
{Phi(t) p(g) PIM}	2482.8424	9.6362
{Phi(g*t) p(g) PIM}	2485.7648	12.5586
{Phi(.) p(g) PIM}	2495.6034	22.3972
{Phi(g) p(g) PIM}	2498.2845	25.0783
{Phi(t) p(t) PIM}	2504.4923	31.2861
{Phi(g*t) p(t) PIM}	2508.0149	34.8087
{Phi(t) p(.) PIM}	2508.5977	35.3915
{Phi(g*t) p(.) PIM}	2511.6602	38.454
{Phi(.) p(t) PIM}	2511.9806	38.7744
{Phi(g) p(t) PIM}	2513.7538	40.5476
{Phi(.) p(.) PIM}	2519.3432	46.137
{Phi(g) p(.) PIM}	2521.2297	48.0235

Models differ in whether or not survival and recapture rates are constant (.), vary over time (t), vary among populations (g) or vary interactively with time and population (g*t). The two top-ranked models (in which survival rates were constant or varied over time) fit the data four times better than the top model in which survival varied among populations (Phi(g*t) p(g*t). AICc = corrected Akaike Information Criteria; delta AICc = disparity between a model and the top-ranked model.

### Body condition

**Figure 3:**
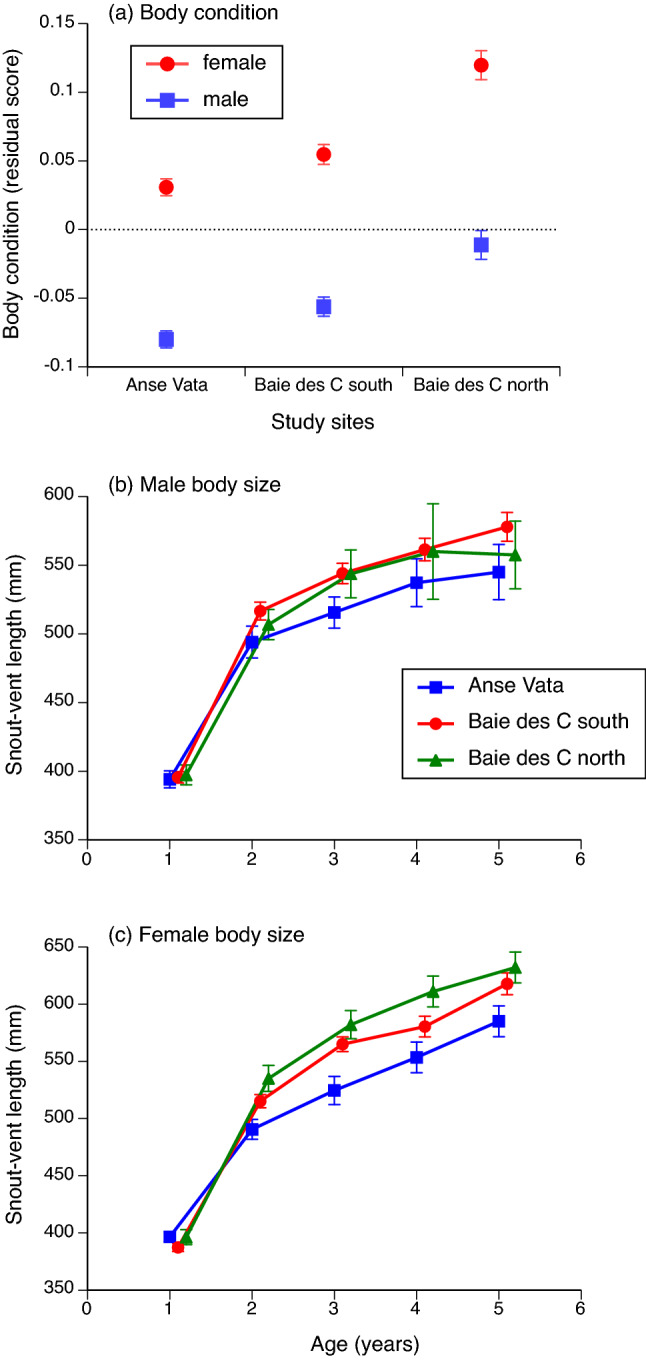
Variation among sites in attributes of turtle-headed sea snakes (*Emydocephalus annulatus*). Panel (**a**) shows body condition (residual score from the general linear regression of ln mass vs. ln snout-vent length, separately for males and females. Panels (**b**) and (**c**) show mean body sizes (snout-vent lengths) and associated standard errors for known-age snakes (based upon initial capture and marking as young-of-the-year). Panel (**b**) shows data for males, whereas panel (**c**) shows data for females.

On average, female snakes were heavier-bodied than were males (main effect of sex *F*_1,2416_ = 553.83, *P* < 0.0001), and snakes from the Baie des Citrons north were heavier-bodied than were snakes from the other sites (main effect of site *F*_2,2416_ = 6.07, *P* < 0.003; interaction sex*site *F*_2,2416_ = 0.36, *P* = 0.70; Fig. 3a).

### Growth curves

Over the first 5 years of life, a snake’s body size increased with age (main effect of age *F*_4,480.5_ = 483.12, *P* < 0.0001) and did so differently in the three sites (interaction age*site *F*_8,480.4_ = 2.73, *P* < 0.006) and between males and females (age*sex *F*_4,480.1_ = 5.44, *P* < 0.0003). The three-way interaction between age, site, and sex was non-significant (*F*_8,480.2_ = 0.51, *P* = 0.85). In both sexes, snakes from Anse Vata grew more slowly than did conspecifics from the other two sites (Fig. 3b,c), although no overall Tukey post-hoc comparisons were significant.

### Reproductive frequency

**Figure 4:**
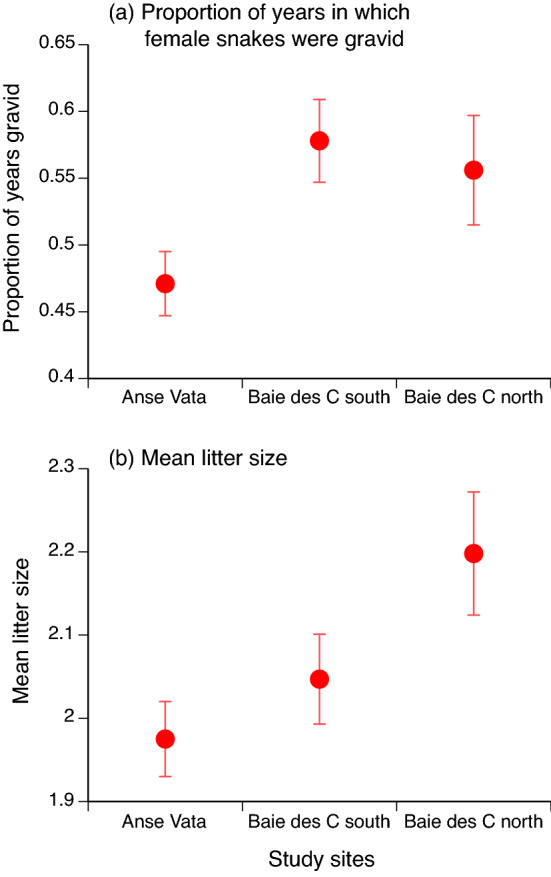
Variation among sites in reproductive output of adult female turtle-headed sea snakes (*Emydocephalus annulatus*). The upper panel (**a**) shows the proportion of years in which female snakes were gravid, based on females caught in at least two years. The lower panel (**b**) shows mean litter sizes, based upon all records. Both graphs show mean values and associated standard errors.

The proportion of years in which multiply-captured females were gravid rather than non-gravid differed among sites (*F*_2,225_ = 14.88, *P* < 0.001) and was higher in larger females (*F*_1,225_ = 28.66, *P* < 0.0001). Maternal body size influenced reproductive frequency in a similar way in all three sites (interaction site*maternal SVL *F*_2,225_ = 0.30, *P* = 0.74). Tukey post-hoc tests show that reproductive frequency was significantly (*P* < 0.05) lower at Anse Vata than at either of the other sites (Fig. 4a).

### Fecundity

Mean litter sizes increased with mean maternal body size (SVL *F*_1,327_ = 29.93, *P* < 0.0001) and were lowest in Anse Vata (*F*_2,327_ = 3.07, *P* < 0.05; Fig. 4b), with a marginally significant interaction term (site*SVL *F*_2,327_ = 3.12, *P* = 0.05; litter sizes increased less rapidly with maternal SVL at Baie des Citrons south than at the other sites). In post-hoc tests, mean litter sizes averaged lower at Anse Vata than in the Baie des Citrons north.

## Discussion

Although our three study sites are very close together, turtle-headed sea snakes rarely move between them^26,27^. That philopatry provides an opportunity for spatial differentiation in life-history traits at a smaller scale than is usually observed, because the abiotic factors that drive resource availability also differ among adjacent bays. Specifically, major differences in compass orientation of the openings to the bays mean that strong winds are far more prevalent at one bay (Anse Vata) than one immediately beside it (Baie des Citrons). The difference in wind strengths between these bays is so consistent that it is frequently mentioned in tourist guides (e.g., https://www.newcaledonia.travel/en/free-things-do-noumea). Annual variation in wind strength is associated with concurrent variation in snake body condition and reproductive output^21^. The likely proximate mechanism for this effect is that winds create strong currents and swell (i.e., choppy seas) in these shallow bays, reducing prey availability by decreased egg production of small demersal fishes^30^ and/or by discouraging snakes from foraging under conditions where scent cues from fish nests are dispersed^31^ and where snakes could be injured by being thrown against coral by the strong swell^21^.

The putative effect of windy conditions on foraging rates is consistent with food-deprived snakes at the windiest site (Anse Vata) being thinner, growing less rapidly, and females reproducing less often and with smaller litters. The effect sizes for some among-site comparisons were substantial (see Figs. 2, 3, 4). Because annual survival rates were similar among sites, variation among individuals in lifetime reproductive success (LRS, = total number of progeny) may be driven largely by variation in reproductive output. Not only did snakes from Anse Vata reproduce less frequently, and with fewer offspring per litter, but females also were smaller at any given age (Fig. 3c). Because both reproductive frequency and litter size increase with maternal body size, the lower growth rate of snakes in Anse Vata would further reduce reproductive output below levels apparent in our (size-corrected) analyses above. Lacking data on ages of most adult females, however, we cannot quantify the magnitude of this additional source of geographic disparity.

Two mechanisms, operating at different timescales, could generate an association between prey availability and snake life-histories. The first is a simple proximate effect, such that the traits of Anse Vata snakes are a direct result of lower feeding opportunities. A second possibility is adaptation, such that each population follows a separate evolutionary trajectory, and snakes in resource-poor environments evolve to reduce their investment of energy into growth and reproduction^32^. The plausibility of that second interpretation is undermined by occasional migration of individuals among populations, because even a low rate of genetic exchange can prevent local adaptation^33^; and also, by the observation that temporal (among-year) as well as spatial variation in life-history traits in this system is linked to wind speeds^21^. Similarly, studies on terrestrial snakes generally interpret site-specific effects as adaptations only for widely-distant (and thus, genetically separate) populations within a single species, or for comparisons among species (but see^34^). In general, variation in life-history traits among individuals from adjacent populations is more likely to be a direct (proximate) effect of resource availability, reflecting phenotypic plasticity in levels of investment into growth and reproduction, rather than a genetically canalised set of adaptations to differing long-term optimal values for such allocation decisions.

Broadly, conclusions from the present analysis support those from an earlier study comparing two of the three sites (Anse Vata vs. Baie des Citrons south^21^) based on a sample of about half the size (and duration of study) as is now available. That earlier analysis reported a similarity in sex ratios between sites, but with Anse Vata snakes thinner and slower-growing (consistent with the present study). The earlier analysis found no significant differences in reproductive frequency or litter size, but such differences are now apparent with a larger sample size, longer duration of study, and inclusion of a third population.

The most striking features of our results, relative to previous research on terrestrial snakes, are (1) the small spatial scale of the life-history divergences; and (2) the inference that life-history variation is driven not by variation in temperature or rainfall, but by exposure to wind. Do these disparities reflect fundamental differences between marine and terrestrial snakes? The first of these patterns (small spatial scale) may largely result from an unusual feature of our study species; most marine snakes are less philopatric than are *E. annulatus*. For example, acoustic tracking of larger snake species in our study areas has shown that greater sea snakes (*Hydrophis major*) travel widely^24^, as do foraging sea kraits (*Laticauda* spp.^35^). A wide-ranging individual can “average out” small-scale variations. Nonetheless, among-population divergence in a phenotypically plastic trait related to foraging (relative head size) has been reported in sea kraits (*Laticauda saintgironsi*), reflecting spatially variable prey sizes combined with strong “home-island” philopatry of adult snakes^36^. Phylogeographic studies have revealed strong population structure even among highly mobile sea snake species (e.g., *Aipysurus laevis*^37,38^). Future work could usefully explore the spatial scale over which individual snakes forage over long time periods (i.e., years), to clarify an individual’s potential sensitivity to spatial variation in prey resources.

The second pattern we have identified – the importance of wind rather than temperature or rainfall as an abiotic driver of life-history variation – is more likely to differ consistently between marine and terrestrial snakes. Water temperatures vary with depth^39^, and sea snakes need access to freshwater from rainfall to maintain their hydric balance^40^; also, females of egg-laying taxa may need to migrate to higher-rainfall sites to obtain access to moist nesting sites^41^. Nonetheless, spatial variation in temperature or rainfall is unlikely to be a primary driver for small-scale spatial variation in life-history traits of adult sea snakes. Instead, parameters such as wind exposure and current strength, combined with seafloor attributes that drive prey availability, are likely to be far more important. Even a small difference in the compass orientation of a bay can massively alter wind speeds and thus, water conditions. Aspects such as water turbidity, swell and currents can modify a snake’s ability to locate prey or mates, and to evade aerial or underwater predators^42^. As a result, biologically important risks and opportunities for marine snakes may vary over smaller spatial scales than for many terrestrial snakes. Contrasts are less clear for temporal variation. For example, the biology of terrestrial snakes often is constrained by diel cycles in temperature^43^ that are far less marked in the ocean; but diel cycles in tidal flow may impose similar constraints^44^.

In summary, our longterm studies of a sea snake species with unusually high philopatry allows us to explore a neglected topic: the magnitude and causes of small-scale spatial variation in life-history traits of these marine predators. Among reef-associated fish on the Great Barrier Reef, demographic parameters such as growth, longevity and mortality differ more markedly along a cross-shelf gradient than along a latitudinal gradient, suggesting that local environmental conditions have a greater influence than does water temperature. For example, angelfish *Centropyge bispinosa* grow faster, attain greater lengths and have higher mortality rates on average on continental than on oceanic reefs^45^. Following the same trend, in four other taxa (the fishes *Chlorurus sordidus, Scarus frenatus*, and *S. niger* and the acanthurid *Acanthurus lineatus*), individuals on outer-shelf reef crests exhibit smaller body sizes, slower growth and reduced life spans than do conspecifics on adjacent mid-shelf reef crests^46^ whereas the reverse is true for the damselfish *Acanthochromis bispinosa*^47^. Feeding rates in marine organisms thus often may be affected by small-scale heterogeneity in water conditions, in turn driven by wind exposure, as much as by larger-scale variation in parameters (such as temperature and rainfall) that drive life-history variation in many terrestrial ectotherms.

## Data Availability

Data can be found in the Dryad Digital Repository at https://www.doi/10.5061/dryad.76hdr7szh.
